# Supplementary material for: Synthesis of magnesium-nitrogen salts of polynitrogen anions
Source: Nat Commun. 2019 Oct 4;10:4515. doi: 10.1038/s41467-019-12530-w (PMC6778147; doi:10.1038/s41467-019-12530-w)
Supplement: Supplementary file 1 — Supplementary Information [file 41467_2019_12530_MOESM1_ESM.pdf]

Supplementary Information

Synthesis of magnesium-nitrogen salts of polynitrogen anions

Laniel *et al*.

Correspondence to: dominique.laniel@uni-bayreuth.de

Supplementary Tables

Supplementary Table 1: Summary of each sample's pressure-temperature path, along with synthesized phases identified upon laser-heating.

Sample Number	Pressure (GPa) before laser-heating	Pressure (GPa) after laser-heating	Measured temperature range (K, ±200)	Synthesized phases
1	28.0*	28.3	2300	Mg_3N_2
	58.1	58.5	1850	β-Mg_2N_4 + MgN_4
2	33.1*	32.8	1900	Mg_3N_2
	43.4	43.7	2000	Mg_3N_2
	52.4	53.5	2300	β-Mg_2N_4 + MgN_4
3	52.2*	52.3	2500	β-Mg_2N_4 + MgN_4
4	52.7*	54.2	2000	β-Mg_2N_4 + MgN_4

*indicates the first pressure at which the sample was laser-heated. Subsequent laser-heating on samples 1 and 2 were performed on the same Mg piece that was previously heated. Between laser-heating pressure steps, the sample was always further compressed. Each sample was decompressed after being laser-heating at their maximum pressure value.

Supplementary Table 2. Full experimental crystallographic data for the Mg_3N_2 compound, compared to the literature values[1] as well as our DFT model.

	**Experimental (single crystal)**	**Experimental (powder data)[1]**	**DFT model**
Chemical formula	Mg_3N_2	Mg_3N_2	Mg_3N_2
Pressure (GPa)	28.3	32.5	28
Space group	*C*2/*m*	*C*2/*m*	*C*2/*m*
a (Å)	12.3498(9)	12.229(9)	12.4475
b (Å)	3.1468(14)	3.157(9)	3.1404
c (Å)	7.6848(4)	7.618(5)	7.7055
β (°)	99.686(7)	99.03(9)	99.8663
V (Å^3)	294.39(14)	290.5	296.759
Fractional atomic coordinates (x y z)	Mg1: 0.82764(15) 0 0.0301(2)<br>Mg2: 0.79509(16) 0 0.3731(3)<br>Mg3: 0.12716(16) 0 0.2854(2)<br>Mg4: 0.46878(17) 0 0.3388(3)<br>Mg5: 0 0.5 0<br>N1: 0.6361(4) 0 0.4853(6)<br>N2: 0.6871(4) 0 0.1366(5)<br>N3: 0.9661(4) 0 0.1854(6)	Mg1: 0.827(6) 0 0.031(4)<br>Mg2: 0.795(1) 0 0.372(1)<br>Mg3: 0.127(8) 0 0.285(6)<br>Mg4: 0.469(1) 0 0.339(6)<br>Mg5: 0 0.5 0<br>N1: 0.636(1) 0 0.484(2)<br>N2: 0.686(4) 0 0.136(9)<br>N3: 0.967(1) 0 0.184(5)	0.8275 0 0.0322<br>0.7937 0 0.3737<br>0.1266 0 0.2840<br>0.4677 0 0.3388<br>0 0.5 0<br>0.6357 0 0.4838<br>0.6871 0 0.1367<br>0.9674 0 0.1836
U_{iso}	Mg1: 0.0074(4)<br>Mg2: 0.0079(4)<br>Mg3: 0.0075(4)<br>Mg4: 0.0094(5)<br>Mg5: 0.0082(6)<br>N1: 0.0100(8)	Mg1: 0.0019(6)<br>Mg2: 0.0077(6)<br>Mg3: 0.0050(9)<br>Mg4: 0.0090(5)<br>Mg5: 0.0025<br>N1: 0.0037(4)	

	N2: 0.0070(7)<br>N3: 0.0074(8)	N2: 0.0023(7)<br>N3: 0.0075(6)	
No. of measured / independent reflections (I ≥ 3σ)	999 / 463 (186)		
R_{int}	0.1199		
Final R indexes (I ≥ 3σ)	R_1 = 0.0803<br>wR_2 = 0.0785		
Final R indexes (all data)	R_1 = 0.1207<br>wR_2 = 0.0912		
No. of parameters	23		

Supplementary Table 3. Full experimental crystallographic data for the MgN_4 compound, compared to the theoretically calculated structure.

	**Experimental (single crystal)**	**DFT model**
Chemical formula	MgN_4	MgN_4
Pressure (GPa)	58.5 GPa	58.5 GPa
Space group	*Ibam*	*Ibam*
a (Å)	3.5860(13)	3.5899
b (Å)	7.526(3)	7.5286
c (Å)	5.1098(17)	5.0551
V (Å^3)	137.90(9)	136.6
Fractional atomic coordinates (x y z)	Mg: 0 0 0.25<br>N1: 0.6584(5) 0.83344(17) 0.5<br>N2: 0.2928(5) 0.3059(5) 0.5	0 0 0.25<br>0.67723 0.8324 0.5<br>0.31042 0.83157 0.5
U_{iso}	N1: 0.0067(2)<br>N2: 0.0062(2)	
U_{aniso} (x y z)	Mg: 0.0064(8) 0.0060(3) 0.0050(3)	
No. of measured / independent reflections ($I \geq 3\sigma$)	413 / 145 (104)	
R_{int}	0.0421	
Final R indexes ($I \geq 3\sigma$)	R_1 = 0.0355;<br>wR_2 = 0.0366	
Final R indexes (all data)	R_1 = 0.0446;<br>wR_2 = 0.0386	
No. of parameters	8	
Mulliken population [e^-]		Mg: + 1.18

		N: -0.29 – -0.30
Bond lengths and populations for shortest bonds		N_1–N_2: 1.317 Å; 0.6 $e^-/Å^3$<br>N_2–N_1: 1.324 Å; 0.8 $e^-/Å^3$<br>Mg–N: 1.962 Å; 0.4 $e^-/Å^3$
Band gap		Small (0.18 eV) direct band gap at W point

Supplementary Table 4. Full experimental crystallographic data for the *β*-Mg_2N_4 compound at 58.5 GPa along with the DFT model.

	Experimental (single crystal)				DFT model		
Chemical formula	*β*-Mg_2N_4				*β*-Mg_2N_4		
Pressure (GPa)	58.5 GPa				58.5 GPa		
Space group	$P2_1/n$				$P2_1/n$		
a (Å)	7.113(5)				7.0436		
b (Å)	5.828(6)				5.84517		
c (Å)	8.800(9)				8.788973		
β (°)	104.00(7)				103.39		
V (Å^3)	354.0(6)				352.01		
Fractional atomic coordinates (x y z)	Mg1:	0.49079(16)	0.7820(2)	0.36972(17)	0.49022	0.78004	0.37017
	Mg2:	0.87494(17)	1.0122(2)	0.65121(19)	0.87512	1.01397	0.65081
	Mg3:	0.27163(17)	-0.0478(3)	0.08387(17)	0.26913	-0.04932	0.08276
	Mg4:	0.73162(16)	1.0462(3)	0.26126(17)	0.73029	1.04401	0.26098
	N1:	0.9146(4)	0.7621(6)	0.4030(4)	0.91472	0.75915	0.402391
	N2:	0.5752(4)	0.2424(6)	0.4128(4)	0.57642	0.24148	0.413907
	N3:	0.5700(4)	0.4601(6)	0.3759(4)	0.56956	0.45831	0.375586
	N4:	1.0672(4)	0.6905(5)	0.5081(4)	1.06662	0.68941	0.507163
	N5:	1.0586(4)	0.7188(6)	0.6557(4)	1.05827	0.72199	0.654191
	N6:	0.2820(4)	0.2132(6)	0.2432(4)	0.28242	0.21213	0.242347
	N7:	0.4332(4)	0.1114(6)	0.3403(4)	0.43202	0.11182	0.337759
	N8:	0.7714(4)	0.8544(6)	0.4546(4)	0.77051	0.85428	0.455001
U_{iso}	Mg1:	0.0100(2)					
	Mg2:	0.0112(3)					

	Mg3: 0.0112(2)<br>Mg4: 0.0106(2)<br>N1: 0.0125(5)<br>N2: 0.0123(5)<br>N3: 0.0112(5)<br>N4: 0.0097(5)<br>N5: 0.0119(5)<br>N6: 0.0104(5)<br>N7: 0.0124(5)<br>N8: 0.0116(5)	
No. of measured / independent reflections (I ≥ 3σ)	2139 / 1018 (507)	
R_{int}	0.0952	
Final R indexes (I ≥ 3σ)	R_1 = 0.0580; wR_2 = 0.0597	
Final R indexes (all data)	R_1 = 0.0809; wR_2 = 0.0660	
No. of parameters	49	

Supplementary Table 5. Full experimental crystallographic data for the *β*-Mg_2N_4 compound at 48.4 GPa along with the DFT model.

	Experimental (single crystal)	DFT model
Chemical formula	*β*-Mg_2N_4	*β*-Mg_2N_4
Pressure (GPa)	48.4	50
Space group	$P2_1/n$	$P2_1/n$
a (Å)	7.161(5)	7.1287
b (Å)	5.875(4)	5.8848
c (Å)	8.811(3)	8.8681
β (°)	103.51(5)	103.64
V (Å^3)	360.4(4)	361.53
Fractional atomic coordinates (x y z)	Mg1: -0.7681(2) 0.5456(3) -0.73938(16)<br>Mg2: -0.6249(2) 0.5131(3) -0.34896(16)<br>Mg3: -0.7295(2) 1.0461(3) -0.41690(15)<br>Mg4: -0.5067(2) 1.2172(3) -0.12896(16)<br>N1: -0.7173(5) 0.7850(6) -0.2579(3)<br>N2: -0.4405(5) 0.2189(6) -0.3444(4)<br>N3: -0.7254(5) 0.3547(6) -0.5445(3)<br>N4: -0.4349(5) 0.1888(6) -0.4927(3)<br>N5: -0.4284(5) 0.5391(6) -0.1236(4)<br>N6: -0.5838(5) 0.2605(6) -0.5960(4)<br>N7: -0.5673(5) 0.8876(6) -0.1604(4)<br>N8: -0.4234(5) 0.7574(6) -0.0856(4)	
U_{iso}	Mg1: 0.0132(3)<br>Mg2: 0.0134(3)<br>Mg3: 0.0131(3)	

	Mg4: 0.0127(3)<br>N1: 0.0131(6)<br>N2: 0.0136(6)<br>N3: 0.0121(5)<br>N4: 0.0130(5)<br>N5: 0.0133(6)<br>N6: 0.0136(6)<br>N7: 0.0151(6)<br>N8: 0.0139(6)	
No. of measured / independent reflections (I ≥ 3σ)	2138 / 1180 (642)	
R_{int}	0.0577	
Final R indexes (I ≥ 3σ)	R_1 = 0.0673; wR_2 = 0.0646	
Final R indexes (all data)	R_1 = 0.0968; wR_2 = 0.0712	
No. of parameters	49	
Mulliken population [e^-]		Mg: +0.8 – 1.2<br>N bonded to two N: -0.2 – -0.3<br>N bonded to one N: -0.7 – -0.8
Bond lengths and populations for the shortest bonds		N-N: 1.31 – 1.33 Å; 0.7 e^- – 0.8 e^-<br>Mg-N: 1.96 – 2.19 Å; 0.2 e^- – 0.5 e^-
Band gap		Indirect band gap between G – Y of 1.2 eV

Supplementary Table 6. Full experimental crystallographic data for the *β*-Mg_2N_4 compound at 39.1 GPa along with the DFT model.

	Experimental (single crystal)				DFT model
Chemical formula	*β*-Mg_2N_4				*β*-Mg_2N_4
Pressure (GPa)	39.1				40
Space group	$P2_1/n$				$P2_1/n$
a (Å)	7.235(1)				7.2410
b (Å)	5.888(2)				5.9383
c (Å)	8.942(3)				8.9730
β (°)	103.89(2)				103.97
V (Å^3)	369.76(18)				374.42
Fractional atomic coordinates (x y z)	Mg1:	0.62529(19)	0.5105(2)	0.3491(2)	
	Mg2:	0.72801(18)	1.0441(2)	0.4181(2)	
	Mg3:	0.76843(18)	0.5468(2)	0.7389(2)	
	Mg4:	0.50806(18)	0.2160(2)	0.1296(2)	
	N1:	0.4408(4)	0.2191(6)	0.3441(5)	
	N2:	0.4369(4)	0.1903(6)	0.4933(5)	
	N3:	0.4296(4)	0.5394(6)	0.1241(5)	
	N4:	0.5691(4)	0.8821(5)	0.1616(5)	
	N5:	0.7175(4)	0.7825(5)	0.2578(4)	
	N6:	0.7287(4)	0.3523(5)	0.5472(5)	
	N7:	0.4272(4)	0.7557(5)	0.0882(5)	
	N8:	0.5842(5)	0.2599(6)	0.5962(5)	

U_{iso}	Mg1: 0.0092(3)<br>Mg2: 0.0093(3)<br>Mg3: 0.0093(3)<br>Mg4: 0.0080(3)<br>N1: 0.0102(5)<br>N2: 0.0105(6)<br>N3: 0.0102(5)<br>N4: 0.0086(5)<br>N5: 0.0077(5)<br>N6: 0.0087(5)<br>N7: 0.0095(5)<br>N8: 0.0129(6)	
No. of measured / independent reflections (I ≥ 3σ)	1889 / 948 (445)	
R_{int}	0.0581	
Final R indexes (I ≥ 3σ)	R_1 = 0.0519; wR_2 = 0.0545	
Final R indexes (all data)	R_1 = 0.0883; wR_2 = 0.0635	
No. of parameters	49	

Supplementary Table 7. Full experimental crystallographic data for the *β*-Mg_2N_4 compound at 35.4 GPa.

	**Experimental (single crystal)**
Chemical formula	*β*-Mg_2N_4
Pressure (GPa)	35.4
Space group	$P2_1/n$
a (Å)	7.3308(8)
b (Å)	6.0011(17)
c (Å)	9.0257(14)
β (°)	104.2811(13)
V (Å^3)	384.79(13)
Fractional atomic coordinates (x y z)	Mg1: 0.49284(16) 0.7834(3) 0.86989(14)<br>Mg2: 0.26788(17) 0.9553(3) 0.23936(14)<br>Mg3: 0.27318(16) -0.0426(3) 0.58214(14)<br>Mg4: 0.37500(16) 0.4938(3) 0.65298(14)<br>N1: 0.5598(4) 0.7805(7) 0.6546(3)<br>N2: 0.5709(4) 0.4616(8) 0.8743(3)<br>N3: 0.5709(4) 0.2477(7) 0.9121(3)<br>N4: 0.4141(4) 0.7373(7) 0.4043(3)<br>N5: 0.2728(4) 0.6472(8) 0.4548(3)<br>N6: 0.5622(4) 0.8095(7) 0.5076(3)<br>N7: 0.4302(4) 0.1167(7) 0.8379(3)<br>N8: 0.2812(4) 0.2195(7) 0.7418(3)
U_{iso}	Mg1: 0.0059(2)<br>Mg2: 0.0069(3)<br>Mg3: 0.0068(3)<br>Mg4: 0.0062(2)

	N1: 0.0055(5)<br>N2: 0.0080(5)<br>N3: 0.0066(5)<br>N4: 0.0054(5)<br>N5: 0.0071(5)<br>N6: 0.0058(5)<br>N7: 0.0064(5)<br>N8: 0.0065(5)
No. of measured / independent reflections ($I \geq 3\sigma$)	2149 / 1208 (664)
R_{int}	0.0768
Final R indexes ($I \geq 3\sigma$)	R_1 = 0.0621; wR_2 = 0.0627
Final R indexes (all data)	R_1 = 0.0966; wR_2 = 0.0696
No. of parameters	49

Supplementary Table 8. Summary of the lattice parameters of the $P2_1/n$ β-Mg_2N_4 phase for datasets with unrefinable atomic positions.

Pressure (GPa)	*a* (Å)	*b* (Å)	*c* (Å)	*β* (°)	*V* (Å^3)	Reflections
31.5	7.328(2)	5.9620(15)	9.034(3)	104.05(3)	382.9(2)	334
22.2	7.325(2)	6.0595(17)	9.209(3)	103.67(3)	397.2(2)	313
16.5	7.590(7)	6.107(9)	9.302(2)	105.19(5)	416.0(7)	140

Supplementary Table 9. Full experimental crystallographic data for the *α*-Mg_2N_4 compound at ambient conditions along with the DFT model.

	**Experimental (single crystal)**	**DFT model**
Chemical formula	*α*-Mg_2N_4	*α*-Mg_2N_4
Pressure (bar)	1	1
Space group	$P2_1/n$	$P2_1/n$
a (Å)	7.5182(9)	7.5720
b (Å)	6.5426(11)	6.6244
c (Å)	13.4431(19)	13.5491
β (°)	130.080(17)	130.32
V (Å^3)	505.95(18)	518.19
Fractional atomic coordinates (x y z)	Mg1: -0.03444(12) -0.26972(14) -0.14194(7)	-0.03836 -0.26499 -0.14440
	Mg2: 0.19484(12) -0.24500(13) -0.43089(7)	0.19594 -0.24871 -0.43084
	Mg3: -0.15418(12) -0.47368(14) -0.39088(7)	-0.15250 -0.47251 -0.38857
	Mg4: -0.05204(12) 0.03340(14) -0.31550(7)	-0.05410 0.02934 -0.31969
	N1: 0.2053(3) 0.2398(3) -0.29135(15)	0.20249 0.24067 -0.29192
	N2: 0.0799(3) -0.2324(3) -0.32480(15)	0.08415 -0.23530 -0.32396
	N3: 0.1944(3) -0.0551(3) -0.10797(15)	0.19631 -0.05750 -0.10868
	N4: -0.3561(3) -0.1649(3) -0.32605(15)	-0.35714 -0.15973 -0.32821
	N5: -0.0018(3) -0.5637(3) -0.18680(15)	0.00011 -0.56001 -0.182650
	N6: 0.1442(3) 0.3256(3) -0.39668(15)	0.14406 0.32638 -0.39693
	N7: -0.3255(3) 0.2339(3) -0.45463(15)	-0.32760 0.23896 -0.45444
	N8: -0.4397(3) -0.2489(3) -0.43838(16)	-0.43931 -0.24941 -0.43905
U_{iso}	N1: 0.0077(3)	
	N2: 0.0078(3)	

	N3: 0.0079(3)<br>N4: 0.0090(3)<br>N5: 0.0090(3)<br>N6: 0.0083(3)<br>N7: 0.0077(3)<br>N8: 0.0083(3)	
U_{aniso}	Mg1: 0.0096(3)<br>Mg2: 0.0080(3)<br>Mg3: 0.0095(3)<br>Mg4: 0.0089(3)	
No. of measured / independent reflections ($I \geq 3\sigma$)	3044 / 1620 (1058)	
R_{int}	0.0525	
Final R indexes ($I \geq 3\sigma$)	R_1 = 0.0470; wR_2 = 0.0499	
Final R indexes (all data)	R_1 = 0.0641; wR_2 = 0.0543	
No. of parameters	69	

Supplementary Figures

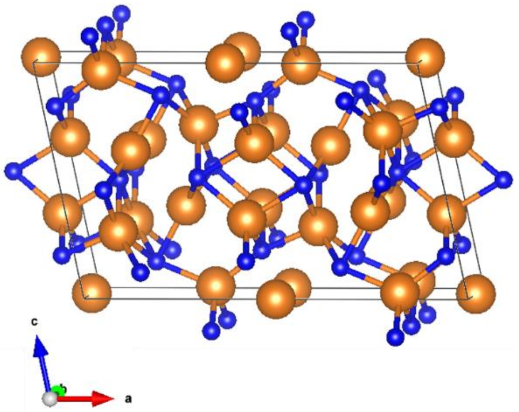

Supplementary Figure 1. Crystal structure of orthorhombic (space group *C*2/*m*) Mg_3N_2 compound at 28.0 GPa. The blue and orange spheres represent N and Mg atoms, respectively.

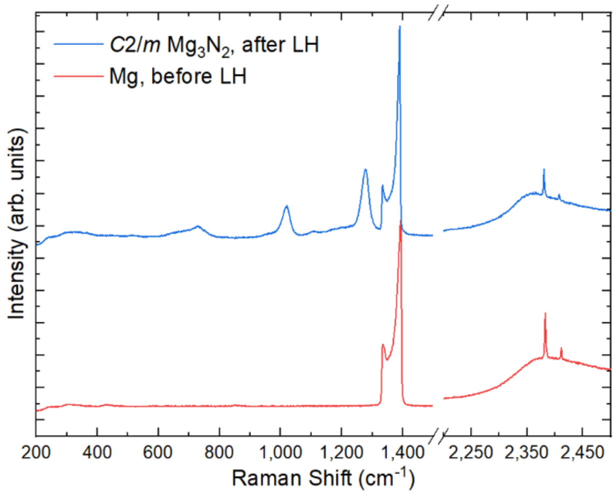

Supplementary Figure 2. Raman spectra before and after laser-heating (LH) the Mg-N_2 sample at 28.0 GPa. The Raman modes detected after heating correspond to Mg_3N_2; the only compound other than the pure elements observed by X-ray diffraction. The intense Raman modes between 1330 and 1400 cm^{-1} belong to the diamond anvils. The modes near 2400 cm^{-1} are produced by pure molecular nitrogen.

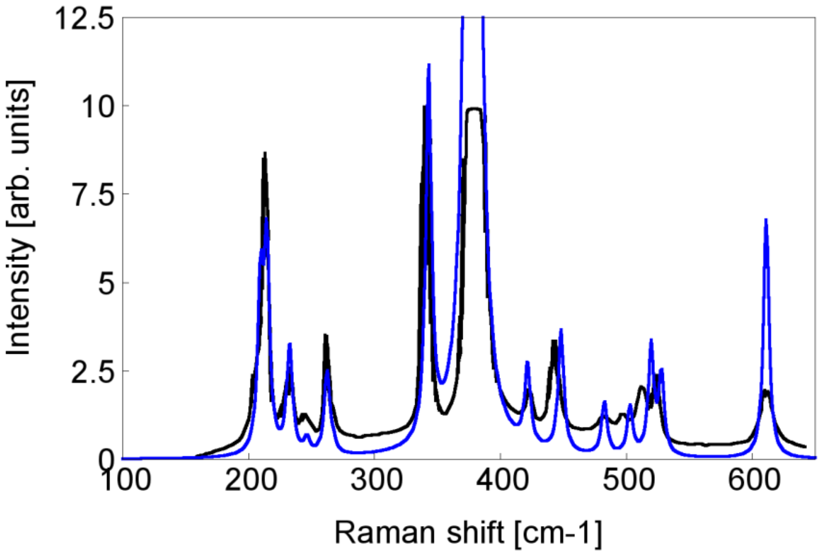

Supplementary Figure 3. Comparison of experimentally determined (black line, ref. 2**) and computed ambient conditions Raman spectrum of cubic Mg_3N_2 (blue line).** The experimental vibron at about 385 cm^{-1} is cutoff due to detector saturation.[2] Clearly, the DFT-GGA-based model describes the frequencies and intensities very well. The frequencies derived from the calculations have been rescaled by 5%.

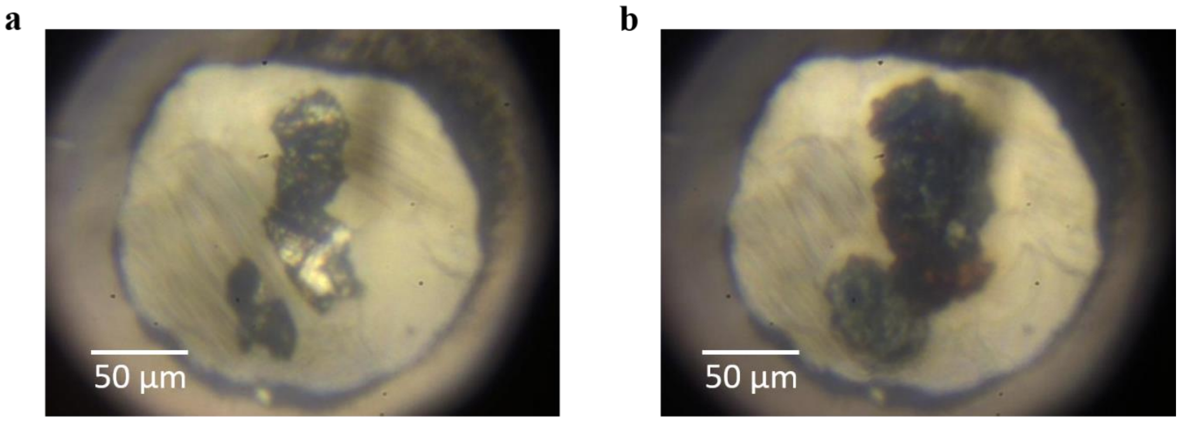

Supplementary Figure 4. Microphotographs of a Mg-N_2 sample (**a**) **before and** (**b**) **after laser-heating at 52.2 GPa.** The pieces of pure metallic Mg are seen to be embedded in a much greater quantity of molecular nitrogen (transparent). The pronounced increase in size after laser-heating attests to a change in volume which reveals, in this case, a chemical reaction.

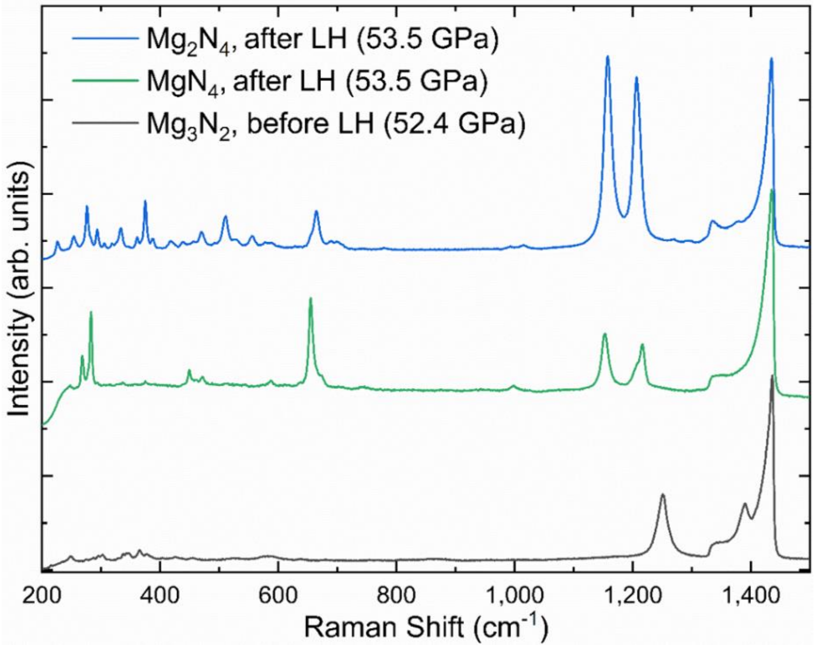

Supplementary Figure 5. Raman spectra of the Mg-N sample before and after laser-heating (LH). Heating allows the *C*2/*m* Mg_3N_2 (black) to react with molecular N_2 and produce MgN_4 (green) and Mg_2N_4 (blue). The pressure increases from 52.4 GPa to 53.5 GPa after laser-heating.

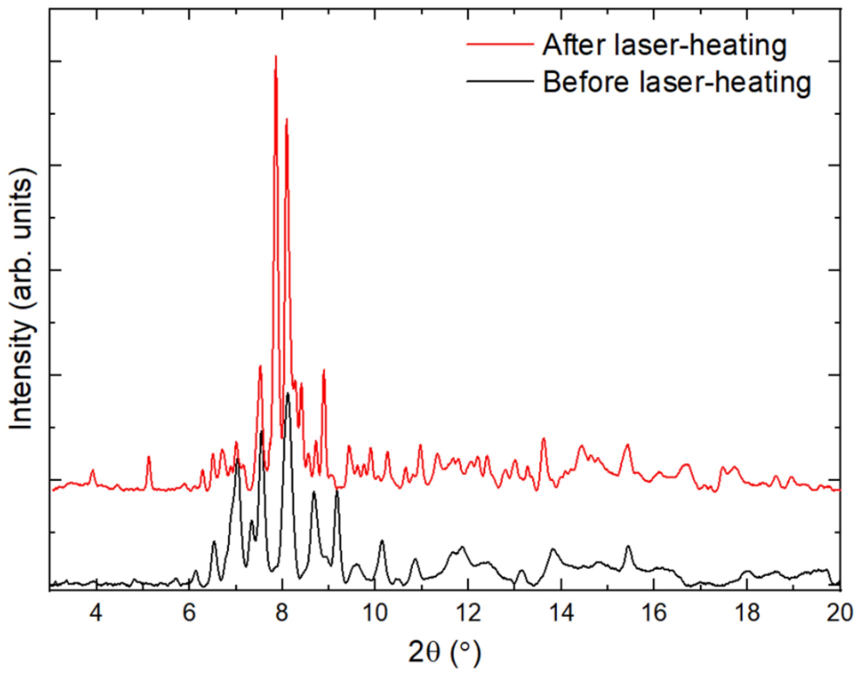

Supplementary Figure 6. Powder X-ray diffraction pattern before (black) and after (red) laser-heating at 58.1 GPa. Before laser-heating, the sample is composed of *C*2/*m* Mg_3N_2, pure ε-N_2 and pure Mg. After laser-heating, new peaks appear—especially easy to spot at low angles—while others disappear. A Le Bail refinement was performed on the diffractogram collected after laser-heating and is shown in Supplementary Supplementary Figure **7**.

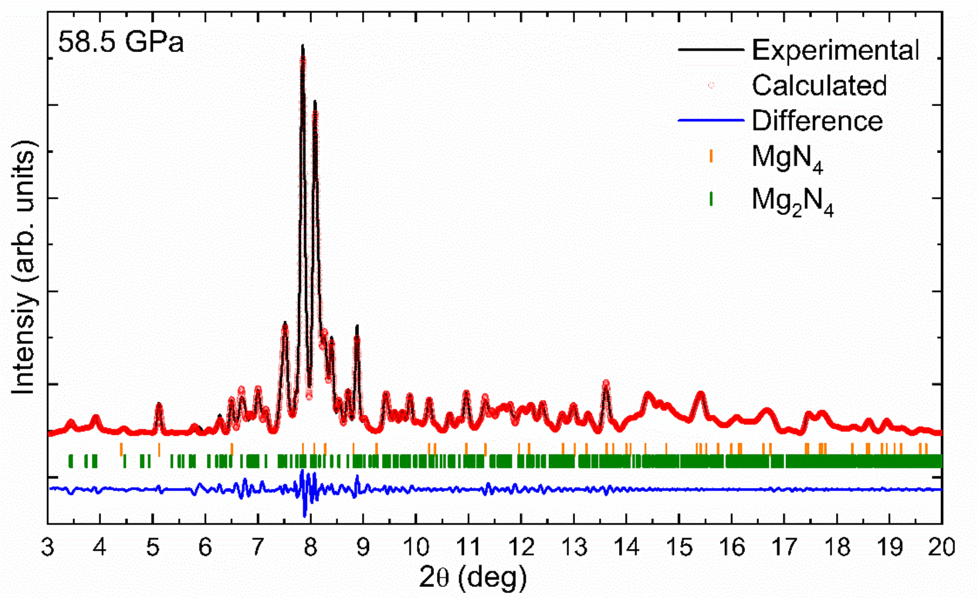

Supplementary Figure 7. A Le Bail refinement using a powder X-ray diffraction pattern obtained at 58.5 GPa. The orange and green tick mark the calculated position of the MgN_4 and Mg_2N_4 diffraction lines. This diffractograms also illustrates the difficulty in identifying the position of the MgN_4 crystallites from a powder pattern. The main MgN_4 diffraction line that can be employed to spot its presence—*i.e.* that is not overlapping with those of Mg_2N_4—is near 5.1° 2θ. If, on account of preferred orientation, this diffraction line is not visible, then the MgN_4 salt can easily go unnoticed. This is hypothesized to be the reason why MgN_4 is not observed by X-ray diffraction at lower pressures despite being observed by Raman spectroscopy.

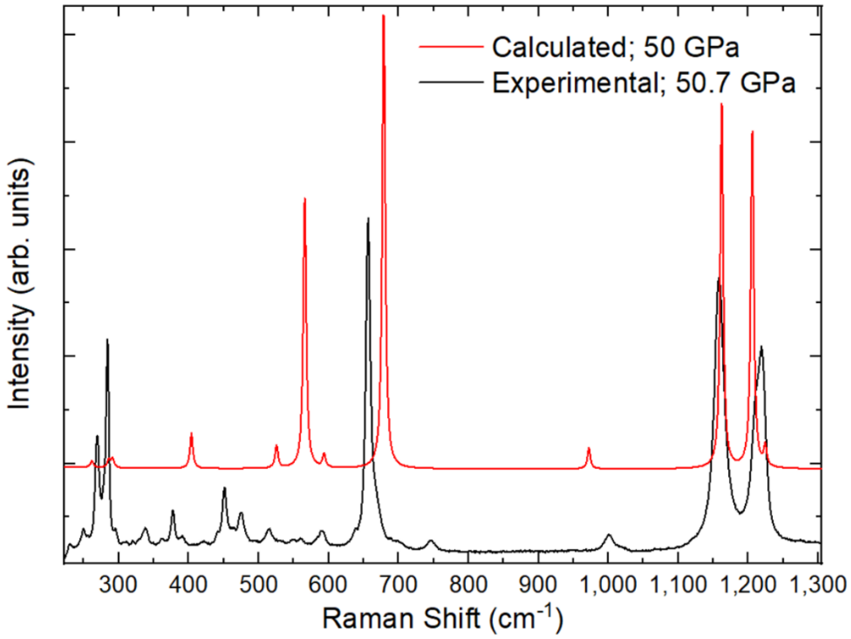

Supplementary Figure 8. Comparison between the calculated and the experimental Raman spectra of MgN_4. The frequencies derived from the calculations have been rescaled by 2%.

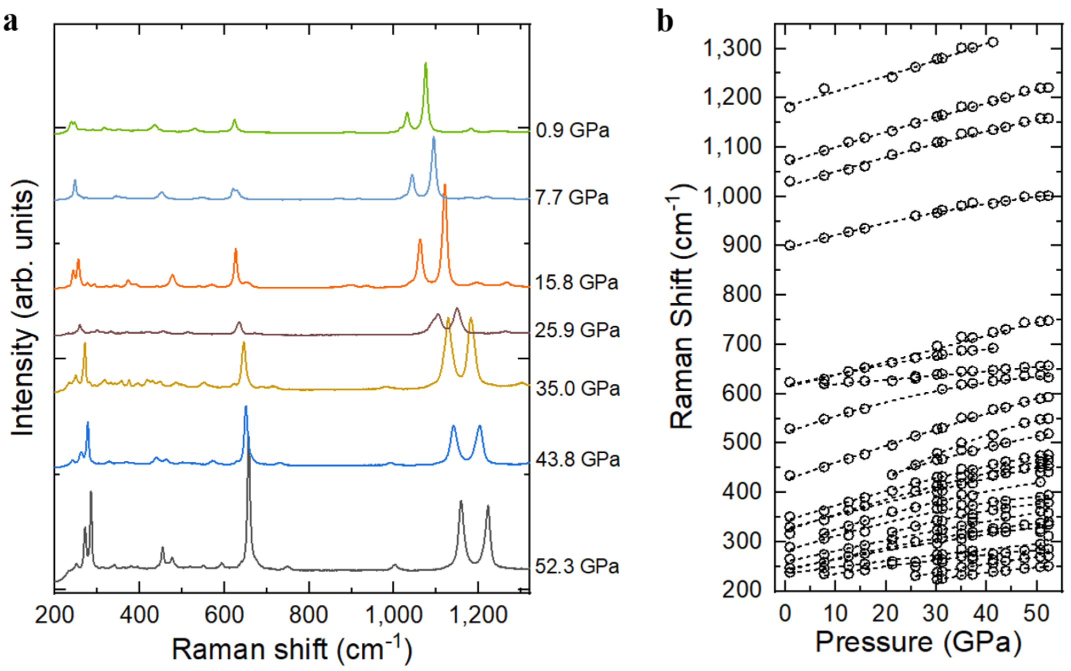

Supplementary Figure 9. Evolution of the Raman modes of MgN_4 with pressure. (**a**) Raman spectra of MgN_4 obtained at various pressures during sample decompression. The spectra are offset along the *y*-axis for clarity. (**b**) Raman shift of the vibrational modes of MgN_4 evolution with pressure. The modes' frequency are observed to shift continuously with pressure, indicative of a lack of phase transition.

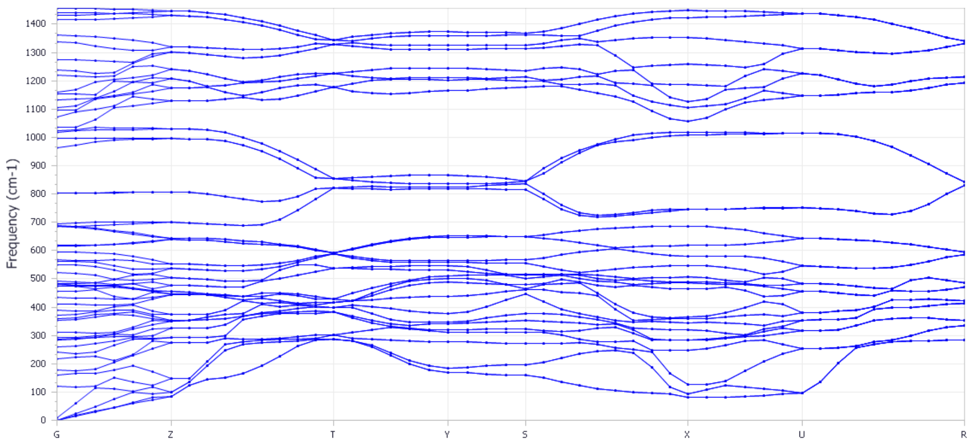

Supplementary Figure 10. Computed phonon dispersion relation for the *Ibam* MgN_4 compound at 50 GPa. The solid is dynamically stable as there are no imaginary frequencies.

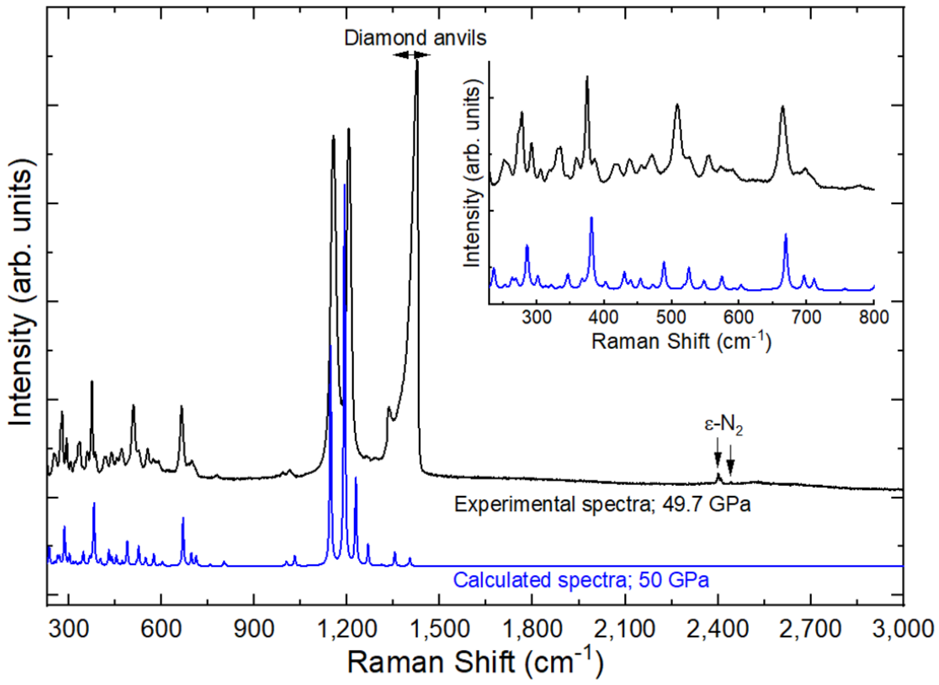

Supplementary Figure 11. Comparison between the experimental and calculated spectra of Mg_2N_4. The calculated spectrum reproduces strikingly well the experimental spectrum. The frequencies derived from the calculations have been rescaled by 3%.

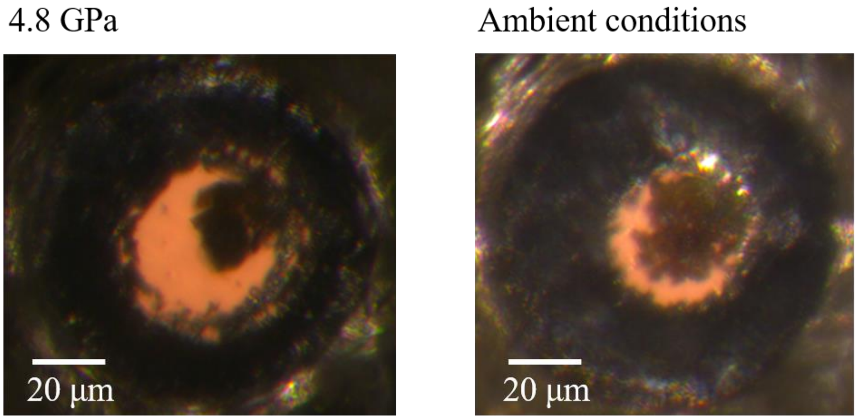

Supplementary Figure 12. Microphotographs of a Mg_2N_4 sample before (left) and after (right) the complete release of pressure. At 4.8 GPa, the DAC was brought in an Ar glovebox and the pressure was brought down to atmospheric pressure as the DAC was opened, allowing for gaseous molecular N_2 to be set free. Afterwards, the DAC was close back and the screws were turned until the experimental cavity noticeably shrunk, sealing it and preventing any exposure to air when brought out of the glovebox. At ambient conditions, the sample is clearly seen to drastically grow in size, which can be explained by the drop in pressure as well as the isosymmetrical $\beta \rightarrow \alpha$ Mg_2N_4 phase transition.

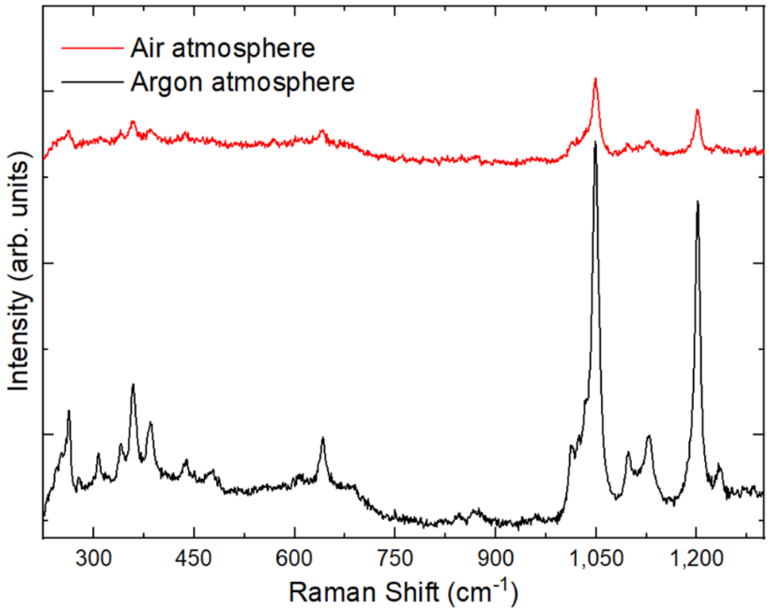

Supplementary Figure 13. Raman spectra of α-Mg_2N_4 sample at ambient conditions under air (red) and argon (black) atmospheres. The spectra in air was obtained immediately after opening the DAC. A few hours later, no signal could be measured.

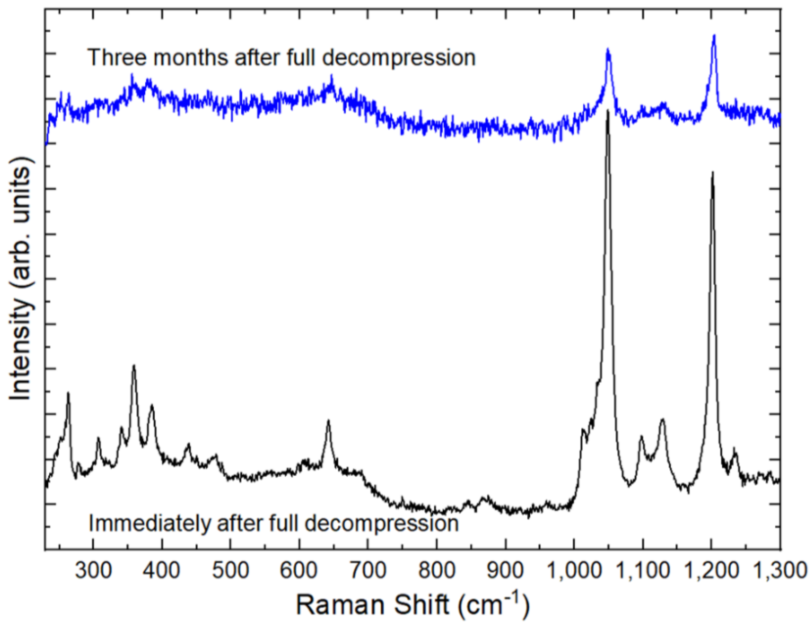

Supplementary Figure 14. Evolution of the Raman spectra of *α*-Mg_2N_4 over time under an argon atmosphere. The black spectrum was obtained immediately after the complete release of pressure. The blue spectrum was acquired three months later, on the same sample. The signal-to-noise ratio is much lower in the blue spectra as it was recorded with a reduced laser power and an acquisition time three times lower to avoid any damage (decomposition) of the solid.

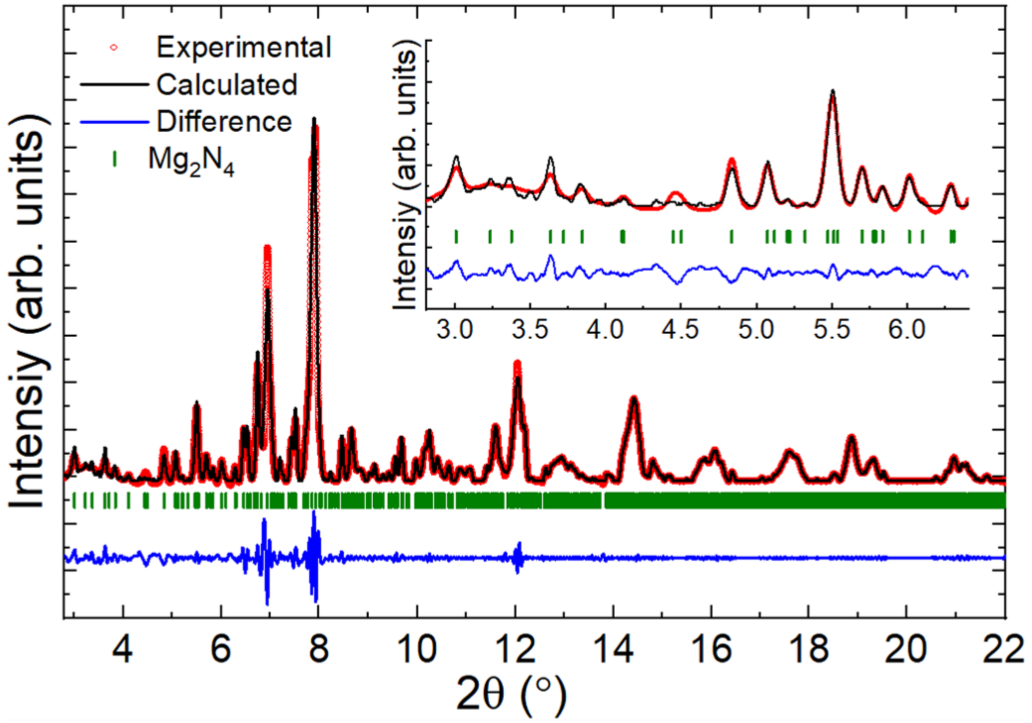

Supplementary Figure 15. A Le Bail refinement employing a powder X-ray diffraction pattern obtained at ambient pressure on a *α*-Mg_2N_4 sample. The green tick lines mark the expected position of the diffraction lines of *α*-Mg_2N_4. (Inset) Enlargement of the low 2θ portion of the refinement.

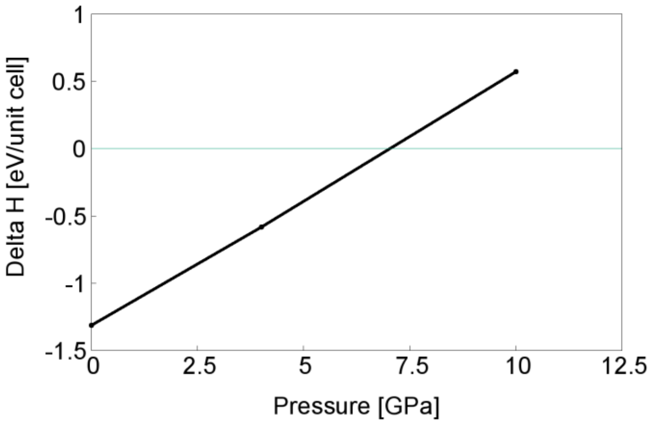

Supplementary Figure 16. Computed enthalpy difference between the *α*- and *β*- polymorph of Mg_2N_4 as a function of pressure. The phase transition is calculated to occur at 7 GPa.

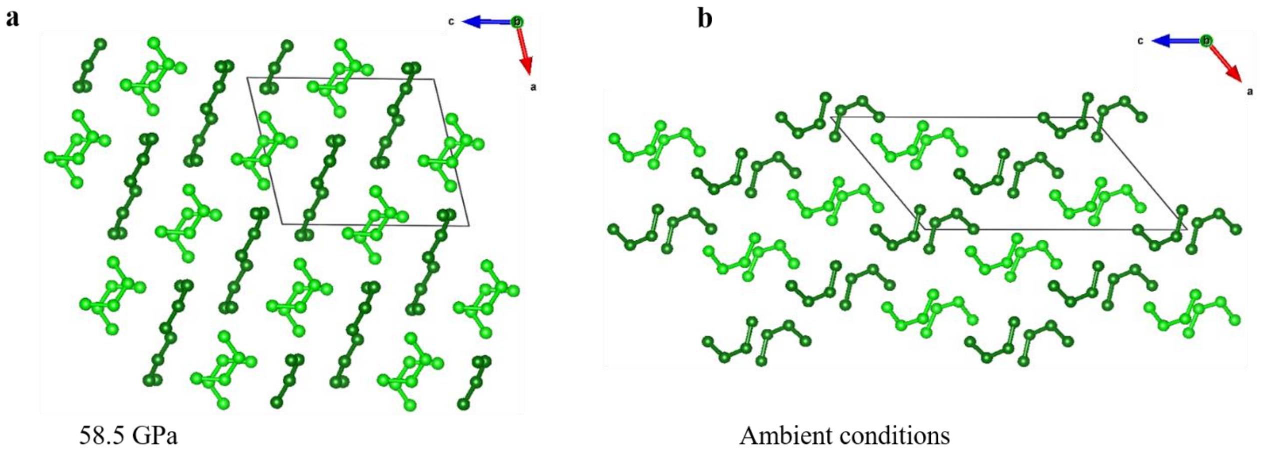

Supplementary Figure 17. Comparison between the N_4^{4-} sublattice (a) of *β*-Mg_2N_4 at 58.5 GPa and (b) of *α*-Mg_2N_4 at ambient conditions. In the *β*-Mg_2N_4 structure, the *a*-N_4^{4-} and *b*-N_4^{4-} entities are arranged in alternating stacks along the [-1 0 1] direction, with the *b*-N_4^{4-} units almost perfectly perpendicular to the *a*-N_4^{4-} units. The *α*-Mg_2N_4 structure shows also shows an alternating stacking of *a'*-N_4^{4-} and *b'*-N_4^{4-} in the [-1 0 1] direction, but in contrast to the *β*-Mg_2N_4 structure the *b'*-N_4^{4-} units are in the same plane as the *a'*-N_4^{4-} units.

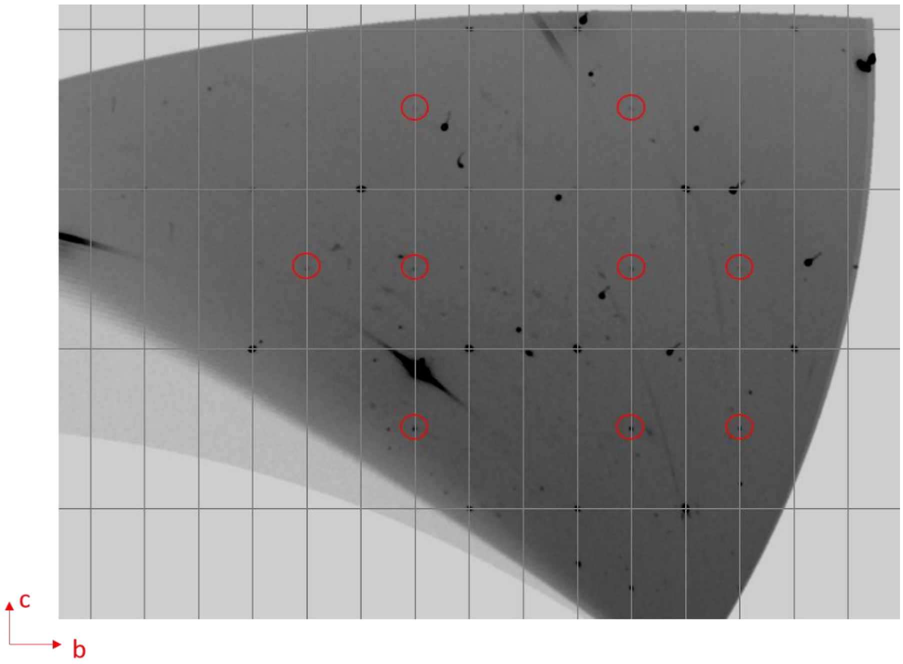

Supplementary Figure 18. Experimental 2kl slice of the reciprocal space of MgN_4 at 58.5 GPa. The grid lines correspond to a *non-doubled c-axis*; with lattice parameters of a = 3.5860(13) Å, b = 7.526(3) Å, c = 2.5549(17) Å. Encircled in red, the distinctly visible reflections corresponding to a doubled c-axis are observed in the middle of the grid lines along the c-direction.

Supplementary Discussion

The Mg_3N_2 phase

The Mg_3N_2 compound was observed after laser-heating magnesium embedded in molecular nitrogen at 28.0, 33.0 and 43.4 GPa. The structure determined from single-crystal X-ray diffraction at 28.0 GPa (see Supplementary Supplementary Figure 1 for a drawing of the structure and Supplementary Table 2 for the full crystallographic parameters) matches the one previously obtained by Hao *et al*. from powder X-ray diffraction measurements.[1] The Raman characterization of this solid is displayed in Supplementary Supplementary Figure 2 and no comparison to literature data is shown as it was not previously measured.

The theoretical results for the high pressure Mg_3N_2 monoclinic polymorph at 28 GPa are in excellent agreement with our experimental findings, as seen in Supplementary Table 2.

We benchmarked our model calculations by comparison of our results to the experimentally determined ambient conditions structure of Mg_3N_2, which adopts the space group *Ia*31 and has a lattice parameter of 9.9528(1) Å.[3] Our calculations gave a = 10.0127 Å, thus showing the often observed slight underbinding in GGA calculations. The experimental Raman spectrum[2] is well reproduced, as shown in Supplementary Figure 3.

Crystallographic data for the MgN_4 salt

The MgN_4 compound adopts an orthorhombic (*Ibam* space group) unit cell with lattice parameters of a = 3.5860(13) Å, b = 7.526(3) Å and c = 5.1098(17) Å at 58.5 GPa. The structure is composed of one Mg (4*a*) and two N (8*j*) atoms on distinct Wyckoff positions (see Supplementary Table 3), and is illustrated in Figure 1. The magnesium atoms are eight-fold coordinated by nitrogen atoms, which are themselves arranged in an exotic planar infinite zigzag N-N chain parallel to the *a*-axis. The N-N distances in the polynitrogen chain alternate between 1.311(3) Å and 1.325(2) Å and,

with typical single and double bonds of about 1.4 Å[4] and 1.25 Å[5–7] in length, respectively, it suggests a bond order between one and two. Assuming a purely ionic Mg-N interaction, the Mg^{2+} atoms would transfer their electronic density to yield N_4^{2-} chain segments. In this fashion, the 20+2 valence electrons of N_4^{2-} are expected to produce four σ-bonds, have four lone electrons pairs and thus six electrons as delocalized π-bonds, compatible with a N-N bond order between one and two. This simple analysis is consistent with the results of the DFT-GGA calculations, which reproduced the MgN_4 structure in great detail.

Insight from DFT calculations on the MgN_4 salt

Our DFT calculations reproduce the alternation of shorter and longer N-N bond lengths in the infinite chains, where unusually, slightly shorter bonds have a marginally higher bond population. The Mulliken charges of the ions are often about half of their formal charge, and this is the case here for Mg (+1.18 e^-), which warrants a description as Mg^{2+}. For comparison, analogous calculations for MgO give Mulliken charges of +0.9 e^- for Mg, -0.9 e^- for O, while for $MgCl_2$ the Mulliken charges are +0.95 e^- for Mg and -0.47 e^- for Cl. In the former, the bond population for 2.12 Å long Mg-O contacts is 1.20 e^-/$Å^3$, while for the 2.53 Å distance of Mg-Cl the bond population is 0.65 e^-/$Å^3$. For a N_2 molecule, the computed bond distance is 1.104 Å and the bond population is 1.32 e^-/$Å^3$.

At 50 GPa, DFT predicts MgN_4 to be elastically stable, with c_{11} = 1212(3) GPa, c_{22} = 764(2) GPa, c_{33} = 481(1) GPa, c_{55} = 36.7(3) GPa, c_{66} = 63(1) GPa, c_{12}=135(1) GPa, c_{13} = 94.0(6) GPa, c_{23}=129.6(3) GPa, and a bulk modulus of K_0 = 310.6(5) GPa.

We have also computed the complete phonon dispersion relation at 50 GPa for the MgN_4 salt, showing it is dynamically stable (Supplementary Supplementary Figure ***10***). From the dispersion curves we calculated the phonon density of states. Whereas in Mg_3N_2 the highest frequency at 50 GPa is of less than 850 cm^{-1}, in MgN_4 at the same pressure the highest frequency is above 1400 cm^{-1}. This showcases the much stronger bonding in MgN_4, as fundamental frequencies are proportional to the strength of the interatomic interactions.

Crystallographic data for the Mg_2N_4 salt

Three Mg_2N_4 samples were studied by X-ray diffraction measurements. The first sample (Mg_2N_4_1) was decompressed from 58.5 GPa with regular pressure steps (58.5, 48.4, 39.1, 22.2, 16.5 and 8.1 GPa) down to ambient conditions, acquiring X-ray diffraction data at each pressure step. The second sample (Mg_2N_4_2) was only characterized by X-ray diffraction at 35.4 GPa and 1 bar, while the third sample (Mg_2N_4_3) was solely investigated by X-ray diffraction at ambient pressure.

Below the pressure of 39.1 GPa, the quality of the Mg_2N_4 crystallites in Mg_2N_4_1 decreased significantly and it was no longer possible to fully resolve the crystalline structure of the salt. However, there were a sufficiently large amount of correlated reflections to allow the determination of the lattice parameters of Mg_2N_4. In Supplementary Table 8 are listed the lattice parameters obtained at various pressure along with the number of reflections employed to refine these values. The employed reflections fit the extinction conditions for the $P2_1/n$ space group of Mg_2N_4. Single crystal data were also collected at a pressure of 8.1 GPa, but the data quality was too poor to even determine the unit cell parameters.

Comment on previous theoretical predictions of Mg-N solids

Calculated convex hulls of the Mg-N system were recently produced from three independent research groups and published in refs. 8–10. Below 60 GPa, the three studies reported similar results, namely compounds with the Mg_3N_2, Mg_2N_3, MgN_2 and MgN_4 stoichiometries becoming stable between ambient and 17 GPa. One set of calculation also predicted a solid with the Mg_5N_4 stoichiometry to be stable from 47 GPa,[8] while another the MgN_{10} compound to be stable from 12 GPa.[10] In comparison, our experimental investigation has resulted in the synthesis of the Mg_3N_2 below 52 GPa and of the Mg_2N_4 (MgN_2 stoichiometry) and MgN_4 solids above that same pressure value. Mg_3N_2 was found to adopt the $C2/m$ space group, in accordance with ref. 8 and previous experimental studies.[1] The MgN_2 and MgN_4 stoichiometries however, were obtained at higher pressures than predicted, which is not an uncommon discrepancy between theory and experiments.[11–13] Regarding the MgN_4 salt, the crystal chemistry (1D N-N chains) was correctly predicted by the calculations, but the here-found space group

Ibam is slightly different than the one predicted, *Cmmm*, and also the *c*-axis was determined by single-crystal X-ray diffraction to be twice what was anticipated. The lattice doubling along the *c*-direction is directly noticeable from the single crystal data unwarping, as shown in Supplementary Supplementary Figure ***18***. Moreover, with the expanded unit cell, over 130 reflections are found to contradict a *C*-centering, with none for an *I*-centering. According to our own DFT calculations, the enthalpy of the *Ibam* structure is comparable to the enthalpy of the *Cmmm* structure, with the former being ~10 kJ/mol lower than the latter, albeit with an uncertainty between 5-10 kJ/mol.

We have also compared the enthalpy of the β-$P2_1/n$ Mg_2N_4 structure at 50 GPa to the enthalpies of the $P6_3/mcm$ and the *Cmcm* MgN_2 structures that had been predicted.[8,9] All were found to be equal within 5 kJ/mol, which is less than the uncertainty of our calculations.

Our results also provide an insight into the predicted Mg-N compounds with the MgN_{10}, Mg_5N_4 and Mg_2N_3 stoichiometry. Since they were not experimentally observed, it can be hypothesized that, like Mg_2N_4 and MgN_4, they are either stable at a higher pressure than expected, or their Gibbs free energy is simply too high compared to that of the other phases, thus prohibiting their synthesis.

Supplementary References

1. Hao, J. *et al.* Structural Phase Transformations of Mg3N2 at High Pressure: Experimental and Theoretical Studies. *Inorg. Chem.* **48**, 9737–9741 (2009).

2. Heyns, A. M., Prinsloo, L. C., Range, K. J. & Stassen, M. The Vibrational Spectra and Decomposition of α-Calcium Nitride (α-Ca3N2) and Magnesium Nitride (Mg3N2). *J. Solid State Chem.* **137**, 33–41 (1998).

3. Partin, D. E., Williams, D. J. & O'Keeffe, M. The Crystal Structures of Mg3N2and Zn3N2. *J. Solid State Chem.* **132**, 56–59 (1997).

4. Eremets, M. I. *et al.* Structural transformation of molecular nitrogen to a single-bonded atomic state at high pressures. *J. Chem. Phys.* **121**, 11296–300 (2004).

5. Schneider, S. B., Frankovsky, R. & Schnick, W. Lithium diazenide high-pressure synthesis and characterization of the alkali diazenide Li2N2. *Angew. Chem. Int. Ed.* **51**, 1873–1875 (2012).

6. Schneider, S. B., Frankovsky, R. & Schnick, W. Synthesis of Alkaline Earth Diazenides MAEN2 (MAE = Ca, Sr, Ba) by Controlled Thermal Decomposition of Azides under High Pressure. *Inorg. Chem.* **51**, 2366–2373 (2012).

7. Auffermann, G., Prots, Y. & Kniep, R. SrN and SrN2: Diazenides by Synthesis under High N2-Pressure. *Angew. Chemie Int. Ed.* **40**, 547–549 (2001).

8. Yu, S. *et al.* Emergence of Novel Polynitrogen Molecule-like Species, Covalent Chains, and Layers in Magnesium–Nitrogen Mg x N y Phases under High Pressure. *J. Phys. Chem. C* **121**, 11037–11046 (2017).

9. Wei, S. *et al.* Alkaline-earth metal (Mg) polynitrides at high pressure as possible high-energy materials. *Phys. Chem. Chem. Phys.* **19**, 9246–9252 (2017).

10. Xia, K. *et al.* Pressure-Stabilized High-Energy-Density Alkaline-Earth-Metal Pentazolate

Salts. *J. Phys. Chem. C* **123**, 10205–10211 (2019).

11. Laniel, D., Weck, G., Gaiffe, G., Garbarino, G. & Loubeyre, P. High-Pressure Synthesized Lithium Pentazolate Compound Metastable under Ambient Conditions. *J. Phys. Chem. Lett.* **9**, 1600–1604 (2018).

12. Laniel, D., Weck, G. & Loubeyre, P. Direct Reaction of Nitrogen and Lithium up to 75 GPa: Synthesis of the Li 3 N, LiN, LiN 2 , and LiN 5 Compounds. *Inorg. Chem.* **57**, 10685–10693 (2018).

13. Laniel, D., Dewaele, A. & Garbarino, G. High Pressure and High Temperature Synthesis of the Iron Pernitride FeN 2. *Inorg. Chem.* **57**, 6245–6251 (2018).
